# Deep Relevance Hashing for Remote Sensing Image Retrieval

**DOI:** 10.3390/s25206379

**Published:** 2025-10-16

**Authors:** Xiaojie Liu, Xiliang Chen, Guobin Zhu

**Affiliations:** 1 School of Remote Sensing and Information Engineering, Wuhan University, Wuhan 430079, China; 2023202130018@whu.edu.cn (X.L.); 2021182130034@whu.edu.cn (X.C.); 2 School of Intelligent Connected Vehicle, Hubei University of Automotive Technology, Shiyan 442002, China

**Keywords:** content-based remote sensing image retrieval (CBRSIR), global hash learning model (GHLM), local hash re-ranking model (LHRM), deep hashing

## Abstract

With the development of remote sensing technologies, the volume of remote sensing data is growing dramatically, making efficient management and retrieval of large-scale remote sensing images increasingly important. Recently, deep hashing for content-based remote sensing image retrieval (CBRSIR) has attracted significant attention due to its computational efficiency and high retrieval accuracy. Although great advancements have been achieved, the imbalance between easy and difficult image pairs during training often limits the model’s ability to capture complex similarities and degrades retrieval performance. Additionally, distinguishing images with the same Hamming distance but different categories remains a challenge during the retrieval phase. In this paper, we propose a novel deep relevance hashing (DRH) for remote sensing image retrieval, which consists of a global hash learning model (GHLM) and a local hash re-ranking model (LHRM). The goal of GHLM is to extract global features from RS images and generate compact hash codes for initial ranking. To achieve this, GHLM employs a deep convolutional neural network to extract discriminative representations. A weighted pairwise similarity loss is introduced to emphasize difficult image pairs and reduce the impact of easy ones during training. The LHRM predicts relevance scores for images that share the same Hamming distance with the query to reduce confusion in the retrieval stage. Specifically, we represent the retrieval list as a relevance matrix and employ a lightweight CNN model to learn the relevance scores of image pairs and refine the list. Experimental results on three benchmark datasets demonstrate that the proposed DRH method outperforms other deep hashing approaches, confirming its effectiveness in CBRSIR.

## 1. Introduction

With the continuous development of observation and sensor technologies, there has been a significant increase in both the quantity and resolution of remote sensing images, making large-scale, high-dimensional remote sensing image archives more common [1]. How to retrieve relevant information from such massive archives of remote sensing images remains a challenge. To address this, content-based remote sensing image retrieval (CBRSIR) has attracted widespread attention as an effective approach to managing large-scale remote sensing data [2]. As a subtask of remote sensing image retrieval (RSIR), CBRSIR aims at searching remote sensing images similar to a query image from a database based on image content, rather than relying on metadata or annotated tags. CBRSIR mainly consists of two core components: feature extraction and similarity measurement.

The complexity of remote sensing image content presents a major challenge in feature extraction for CBRSIR. Specifically, the objects that define the image’s category may comprise only a small portion of the image, limiting their prominence in the image’s content. Additionally, the objects are often scattered across the image, and objects from different categories may overlap or interfere with each other, which increases the difficulty of distinguishing between them during feature extraction. Therefore, how to effectively integrate both global and local information for precise retrieval is a key issue in CBRSIR.

In the feature extraction step, the feature extractor converts remote sensing images into visual representations. The extracted features of remote sensing images can be categorized into three types. The first category includes low-level features, such as edges [3], colors [4,5], and textures [6], which are hand-crafted based on the basic pixel information of the images. These features were widely used in the early remote sensing image retrieval systems. The second category comprises mid-level features, such as bag of visual words (BoVW) [7] and vector of locally aggregated descriptors (VLAD) [8]. These features aggregate and encode low-level features to generate more robust and discriminative image representations. Compared to low-level features, mid-level features demonstrate greater robustness to image transformations, such as rotation, scaling, and translation, thereby improving their effectiveness in retrieval tasks that require more sophisticated semantic representations [9]. The third category focuses on high-level features extracted by a convolutional neural network (CNN), which have been widely used in CBRSIR [10]. CNN models can learn discriminative features from large-scale remote sensing images to improve retrieval performance.

After feature extraction, the similarity between the query image and database images is computed to generate a ranking list. Similarity measurements can be categorized into two types: real-valued feature-based methods and hashing-based methods [2]. Methods relying on real-valued features typically use continuous features to compute distances between images. Although these approaches can effectively capture subtle differences between features, their computational costs are prohibitively high, especially when processing high-dimensional deep features in large-scale remote sensing databases. To overcome this, hashing-based approaches have been proposed. The main idea of hashing is to map continuous features to compact binary codes and measure similarity by calculating the Hamming distance between the hash codes using XOR operations, which reduces both storage requirements and computational complexity.

With the rapid development of deep learning, deep hashing methods have made great progress in CBRSIR. For example, Li et al. [11] proposed a large-scale remote sensing image retrieval method based on deep hashing neural networks (DHNNs), which combines deep feature learning with hash mapping and achieves satisfactory performance. Li et al. [12] proposed the quantized deep learning hash (QDLH) framework, which binarizes weights and activation functions to reduce storage space and computational resources.

**Figure 1:**
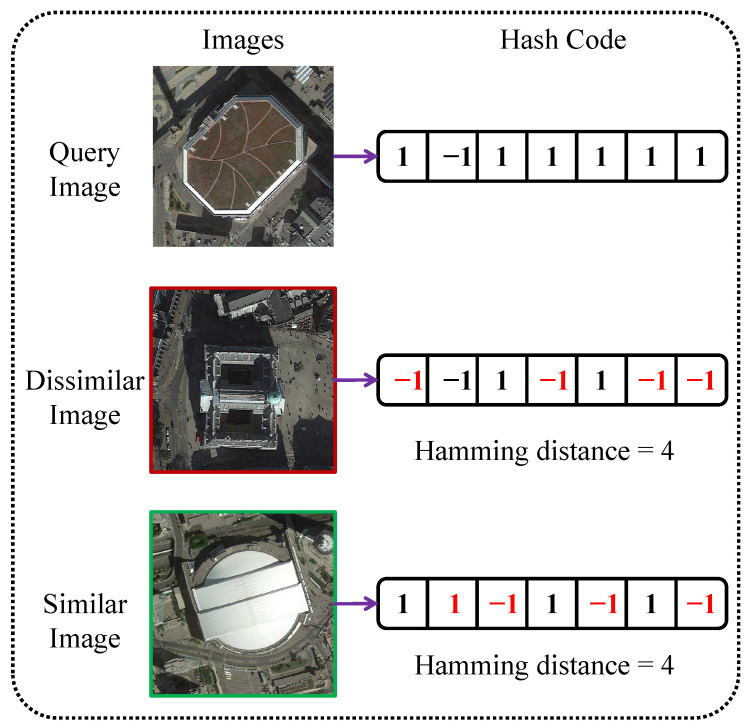
Examples of difficult pairs and retrieval confusion. The middle image belongs to a different category than the query but shares a similar background. The bottom image is from the same category as the query but appears visually different. Both images have the same Hamming distance, making them challenging to distinguish during retrieval.

However, the RS hashing methods mentioned above do not address the varying difficulties among image pairs or the ambiguity in retrieval results caused by equal Hamming distances, as illustrated in Figure 1. Moreover, most existing methods only perform weighting at the batch level, typically assigning uniform or class-based weights to all similar or dissimilar pairs within a mini-batch [13,14,15], without considering the difficulty of individual pairs. Such strategies may alleviate sample quantity imbalance, but they fail to consider the difficulty of individual pairs, leading to suboptimal training. Due to the discrete nature of Hamming distance and the low inter-class variation yet high intra-class variation commonly observed in RS images [14], it is common for multiple semantically different images to have the same distance to a query image. This makes fine-grained ranking particularly important.

To address the aforementioned challenges, we propose a novel deep relevance hashing (DRH) method for remote sensing image retrieval. DRH consists of two key components: the global hash learning model (GHLM) and the local hash re-ranking model (LHRM). GHLM is designed to learn hash codes while considering the difficulty of image pairs. It comprises a backbone network, a hash layer, and a classification layer. The backbone network extracts features from remote sensing images, where global features from the fully connected layer are used to assess pairwise difficulty and weight the similarity loss during training. Meanwhile, local features from the convolutional layer are utilized to construct a relevance matrix for LHRM. The hash layer generates hash codes for initial ranking, while the classification layer incorporates semantic information into the learning process. LHRM adopts a lightweight CNN model to predict relevance scores between the query and database images based on the relevance matrix, which are then utilized to refine the initial ranking. The main contributions of this paper can be summarized as follows:

1. We propose a global hashing learning model (GHLM) to explore image features from different perspectives. The GHLM incorporates a weighted similarity loss to evaluate the differences between easy and difficult image pairs, thereby improving the discriminative capacity of the generated hash codes.
2. We design a local hashing reranking model (LHRM) to refine the initial retrieval results. The LHRM predicts relevance scores to reduce confusion among images with identical Hamming distances, further enhancing retrieval precision and robustness.
3. We conduct extensive experiments on three benchmark datasets. The results demonstrate that the proposed method consistently outperforms other competitive approaches.

The remaining parts of this paper are organized as follows. Section 2 provides a review of the related work. Section 3 describes the proposed method in detail. Section 4 presents the experimental results and discussions. Finally, Section 5 concludes the paper.

## 2. Related Work

### 2.1. Hash Function Learning

In this section, we will present a detailed literature review of three main categories of hash function learning: unsupervised hashing, semi-supervised hashing, and supervised hashing. Unsupervised hashing focuses on learning compact binary codes without relying on labeled data. For example, Weiss et al. [16] proposed spectral hashing to perform semantic hashing by formulating the problem as a relaxed graph partitioning task, where hash codes are derived by thresholding eigenvectors of the graph Laplacian. Gong et al. [17] formulated learning binary codes as an orthogonal Procrustes problem and proposed iterative quantization (ITQ), a simple and efficient alternating minimization method to minimize quantization error. Liu et al. [18] proposed a novel graph-based hashing method that automatically discovers the neighborhood structure inherent in the data. Due to the lack of semantic information, unsupervised methods cannot adequately capture the underlying data relationships, resulting in limited performance.

In contrast to unsupervised hashing, semi-supervised hashing can learn a hash function by utilizing both labeled and unlabeled training samples, thereby enhancing the capacity of the learned codes. For example, Wang et al. [19] proposed a semi-supervised hashing (SSH) framework that minimizes empirical error on the labeled data while incorporating an information-theoretic regularizer over both labeled and unlabeled samples. Wang et al. [20] proposed a novel semi-supervised hashing method that preserves pairwise constraints for both low-dimensional embeddings and binary codes by representing data points with cluster centers. Zhang et al. [21] proposed a semi-supervised deep hashing method, in which a semi-supervised loss is employed to concurrently preserve both the semantic similarity and the intrinsic structure of the data. Yan et al. [22] proposed a semi-supervised hashing method named deep hashing with a bipartite graph (BGDH), which constructs a bipartite graph to capture data structure and generate embeddings. Compared to unsupervised hashing, semi-supervised hashing can learn hash codes from both unlabeled data and a limited amount of labeled data. However, its performance is influenced by the quality of the labeled samples.

As labeled data becomes more accessible in certain domains, supervised hashing methods have been widely explored to enhance retrieval accuracy. For example, Liu et al. [23] proposed a kernel-based supervised hashing model that achieves high-quality hashing with low training cost by optimizing the equivalence between code inner products and Hamming distances. Shen et al. [24] proposed a supervised hashing framework that introduces an auxiliary variable to reformulate the optimization objective, making it solvable through regularization. Kang et al. [25] proposed column sampling-based discrete supervised hashing (COSDISH), which leverages column sampling and alternating optimization for efficient image retrieval. In [26], Cao et al. proposed deep Cauchy hashing (DCH), a deep hashing model that uses pairwise cross-entropy loss based on the Cauchy distribution for retrieval in Hamming space. Peng et al. proposed a Swin Transformer-based supervised hashing (SWTH) method [27], which leverages the Swin Transformer as the feature extraction backbone to capture global contextual information and learn compact hash codes.

### 2.2. Hashing for Remote Sensing Image Retrieval

Hashing methods for remote sensing image retrieval can be broadly categorized into non-deep and deep methods. Non-deep hashing methods typically rely on traditional features to generate binary codes for image retrieval. For example, Chaudhuri et al. [28] proposed an unsupervised graph-theoretic approach for region-based remote sensing image retrieval. Demir et al. [29] proposed two kernel-based nonlinear hashing methods for image retrieval in large-scale remote sensing archives. Reato et al. [30] proposed an unsupervised method that uses primitive-cluster sensitive multi-hash codes and performs better compared to traditional single-hash methods. Fernandez-Beltran et al. [31] introduced the probabilistic latent semantic hashing (pLSH) model, which uses probabilistic topic models to capture the hidden semantic patterns of images. Over the past decades, non-deep hashing methods were widely used in RSIR. However, handcrafted features may not fully capture the complex semantic information of remote sensing images, thereby leading to suboptimal retrieval results.

With the development of deep learning, many deep hashing methods for remote sensing image retrieval have been proposed. Tang et al. [32] proposed a novel semi-supervised deep adversarial hashing (SDAH) method that integrates a residual auto-encoder (RAE) and two multi-layer networks for RSIR within a generative adversarial framework. Han et al. [33] developed a cohesion-intensive deep hashing model that employs a weighted loss strategy to enhance the cohesiveness of hash codes within each class and address data imbalance. Shan et al. [34] proposed hard probability sampling hashing (HPSH), a deep hashing method that combines hash code learning with hard probability sampling to select informative and discriminative samples. Roy et al. [35] proposed a metric-learning-based hashing network that leverages a pretrained deep neural network for intermediate feature representation without retraining and learns a semantic-based metric space optimized for retrieval. Shan et al. [36] introduced deep hashing using proxy loss (DHPL), a proxy-based hash retrieval method combining hash code learning with proxy metric learning in a convolutional neural network. Liu et al. [15] proposed a deep hashing method called deep hashing based on classification label (DHCL), which utilizes classification labels as semantic cues to enhance retrieval performance. Zhang et al. [13] proposed a novel deep attention hashing method (DAH) for RSIR. A channel-spatial joint attention mechanism was employed to extract discriminative features. In the retrieval phase, a distance-adaptive ranking strategy based on category-weighted Hamming distance was adopted to fully leverage category probability information. Zhao et al. [37] proposed multiscale context deep hashing to effectively integrate multiscale contextual information into deep hashing frameworks. Zhou et al. proposed a deep global semantic structure-preserving hashing (DGSSH) framework [38], which leverages a Swin Transformer for feature extraction and introduces a corrective triplet loss to enhance hashing performance.

In this paper, we focus on supervised deep hashing for CBRSIR. Our proposed DRH method fully considers the varying difficulty of image pairs by integrating a weighted similarity loss that prioritizes more challenging pairs during training. Additionally, it employs LHRM to refine retrieval results and reduce confusion. These enhancements further improve retrieval accuracy and robustness.

## 3. Proposed Method

**Figure 2:**
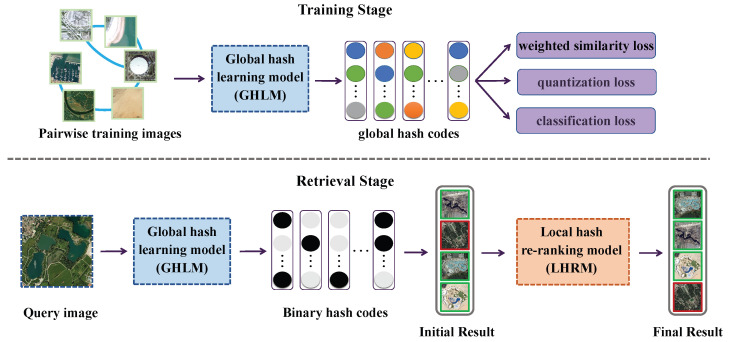
The framework of the proposed deep relevance hashing (DRH) method. During training, the global hashing learning model (GHLM) learns global hash codes by optimizing a combination of multiple losses. During retrieval, the trained GHLM extracts binary hash codes from remote sensing images for initial ranking, and the local hashing reranking model (LHRM) is applied to refine the retrieval results.

In this section, we will provide a detailed introduction to the proposed deep relevance hashing method. First, we introduce the definition of hash code learning. Then, we present the GHLM and the weighted pairwise similarity loss. Finally, we describe the LHRM. The overall framework of the proposed DRH method is illustrated in Figure 2.

### 3.1. Problem Definition

The aim of deep hashing is to generate compact binary codes for images based on their semantic information. Assume that the RS image dataset contains *N* RS images ${\left\{I_{i}\right\}}_{i=1}^{N}$ and their corresponding *N* labels ${\left\{y_{i}\right\}}_{i=1}^{N}$. Here, $y_{i}\in {\left\{0,1\right\}}^{c}$ represents the label of the *i*-th image $I_{i}$, where *c* is the number of classes. Let $S=\{s_{ij}\}$ denote the pairwise similarity matrix. For a pair of images $I_{i}$ and $I_{j}$, if they belong to the same category ($y_{i}=y_{j}$), then $s_{ij}=1$; otherwise, $s_{ij}=0$. The goal of deep hashing with pairwise labels is to find a nonlinear mapping function $H:\left\{I_{i}\right\}\to b_{i}\in {\left\{-1,1\right\}}^{K}$, which can encode each image $I_{i}$ into compact binary hash codes $b_{i}$ while preserving the similarity of images. If two remote sensing images belong to the same category, the distance between their binary hash codes should be minimized and vice versa.

### 3.2. Global Hash Learning Model

**Figure 3:**
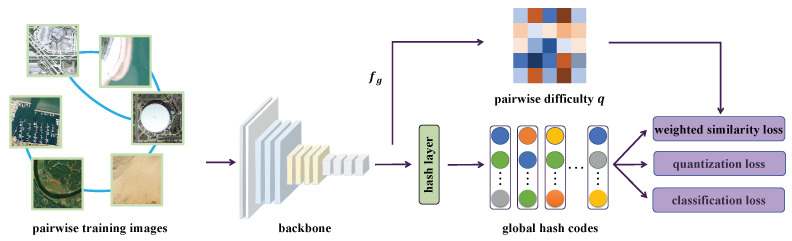
Overview of the global hash learning model (GHLM), which includes a backbone network for feature extraction and a hash layer to generate compact binary codes. The training is optimized by three loss functions: classification loss, quantization loss, and notably, a pairwise similarity loss that assigns weights based on the difficulty of image pairs, evaluated using the global features extracted by the backbone network.

The framework of GHLM is illustrated in Figure 3. The following sections detail its network structure and loss function.

#### 3.2.1. Network Structure

GHLM consists of three main components: a backbone network, a hash layer, and a classification layer. The backbone network extracts features from RS images, the hash layer converts these features into compact binary hash codes, and the classification layer maps the hash codes to labels.

To extract representative features from remote sensing images, we adopt VGG11 [39] as the backbone network, which consists of eight convolutional layers ($conv_{1}$–$conv_{8}$) and two fully connected layers ($fc_{9}$–$fc_{10}$). To adapt it for hash learning, we replace the classifier layer ($fc_{11}$) with a hash layer containing *K* hidden units to generate compact hash codes. The output of $fc_{10}$ serves as the global feature representation $f_{g}^{i}$ for each image $I_{i}$, which is then fed into the hash layer.

The hash layer maps $f_{g}^{i}$ into a *K*-dimensional continuous feature vector $z_{i}\in R^{K}$, which is subsequently binarized using the sign function:

(1) $$b_{i}=sign\left(z_{i}\right).$$

Since the sign function poses an optimization challenge due to its non-differentiability, we approximate it during training using the hyperbolic tangent (tanh) function:

(2) $$h_{i}=\tanh\left(z_{i}\right).$$

To enhance the semantic representation of the learned hash codes, we add a classification layer after the hash layer to predict the label $y_{i}$ of the image $I_{i}$:

(3) $$y_{i}=\omega^{T}h_{i}+b,$$

where ω and *b* denote the parameters of the classification layer and are updated through the backpropagation of the classification loss.

#### 3.2.2. Loss Function

As depicted in Figure 3, the hash learning process is optimized using three loss components: pairwise similarity loss $L_{sim}$, classification loss $L_{class}$, and quantization loss $L_{quan}$. $L_{sim}$ preserves the relative similarity between remote sensing images in the hash space. $L_{class}$ enhances the discriminative power of real-valued hash codes. $L_{quan}$ enforces the hash codes to be closer to binary values, i.e., either −1 or 1. The overall loss function is formulated as:

(4) $$L_{GHLM}=L_{sim}+\alpha L_{class}+\beta L_{quan},$$

where α and β are the hyperparameters that balance the loss terms. Next, we will describe each term in $L_{GHLM}$ in detail.

The pairwise similarity loss $L_{sim}$ is used to learn discriminative hash codes by minimizing the Hamming distance between similar pairs while maximizing it for dissimilar pairs. Given an image pair $\left(I_{i},I_{j},s_{ij}\right)$, the conditional probability of the similarity label $s_{ij}$ given the corresponding hash codes $h_{i}$ and $h_{j}$ is defined as:

(5) $$p\left(s_{ij}|h_{i},h_{j}\right)=\left\{\begin{array}{cc} \sigma (\Omega_{ij}),\; & \mathrm{if}\;s_{ij}=1,\\ 1-\sigma (\Omega_{ij}),\; & \mathrm{if}\;s_{ij}=0, \end{array}\right.$$

where $\Omega_{ij}=\frac{1}{2}h_{i}^{T}h_{j}$, and $\sigma \left(\Omega_{ij}\right)=\frac{1}{1+e^{-\Omega_{ij}}}$. By minimizing the negative log-likelihood over all training pairs, the pairwise similarity loss is formulated as:

(6) $$L_{sim}=-\sum_{s_{ij}\in S}\log p\left(s_{ij}|h_{i},h_{j}\right)=\sum_{s_{ij}\in S}\left(1+e^{-\Omega_{ij}}\right)-s_{ij}\Omega_{ij},$$

where *S* represents the set of similarity labels.

Inspired by Focal Loss [40], we introduce a difficulty-aware weighting scheme to further enhance pairwise supervision. Given that similar deep features facilitate hash code learning [41], we define a difficulty factor *q* for each image pair based on the global feature $f_{g}$ extracted from the backbone network. Specifically, we compute *q* using a contrastive-like formulation:

(7) $$\begin{array}{cc} q_{ij} & =\frac{1}{D}s_{ij}\left|\right|f_{n}^{\left(i\right)}-f_{n}^{\left(j\right)}{\left|\right|}_{2}^{2}\\ \; & \;+\frac{1}{D}\left(1-s_{ij}\right)max(m-||f_{n}^{\left(i\right)}-f_{n}^{\left(j\right)}{\left|\right|}_{2}^{2}{,0)}^{2}, \end{array}$$

where *D* is the dimension of $f_{g}$, and $f_{n}$ is obtained by applying an element-wise sigmoid function to $f_{g}$ to normalize the magnitude of *q*. The first term ensures that for similar pairs, a smaller distance corresponds to lower difficulty, while a larger distance indicates greater difficulty. The second term adjusts dissimilar pairs, increasing difficulty when their distance is smaller than *m* and decreasing it otherwise.

By incorporating difficulty-aware weighting into the pairwise similarity loss, the final difficulty-weighted loss function is formulated as:

(8) $$L_{sim}=\sum_{s_{ij}\in S}e^{q_{ij}}\left(1+e^{-\Omega_{ij}}-s_{ij}\Omega_{ij}\right).$$

The classification loss $L_{class}$ enhances the discriminative power of real-valued hash codes. Given the predicted class probability ${\hat{y}}_{i}$ for an image $I_{i}$, it is formulated as:

(9) $$L_{class}=-\frac{1}{N}\sum_{i}^{N}\left(y_{i}log\left({\hat{y}}_{i}\right)\right),$$

where *N* is the number of training images.

The quantization loss $L_{quan}$ enforces hash codes to be close to −1 or 1, thereby minimizing the gap between continuous and discrete hash codes. It is defined as:

(10) $$L_{quan}=\frac{1}{N}\sum_{i=1}^{n}\left|\right|b_{i}-h_{i}{\left|\right|}_{2}^{2}.$$

### 3.3. Local Hash Re-Ranking Model

**Figure 4:**
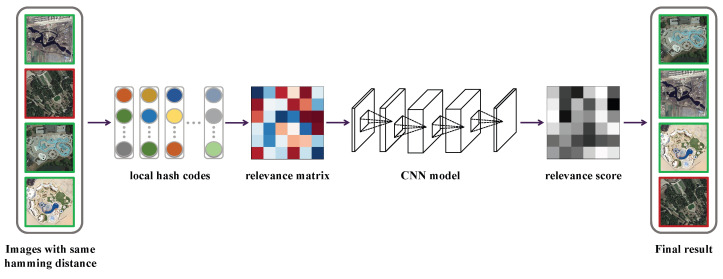
Framework of LHRM.

The architecture of the local hash re-ranking model (LHRM) is shown in Figure 4. First, we construct a relevance matrix based on the initial ranking list. A CNN model is then trained to predict relevance scores, which are used to re-rank the results and obtain the final ranking.

#### 3.3.1. Training Stage

**Figure 5:**
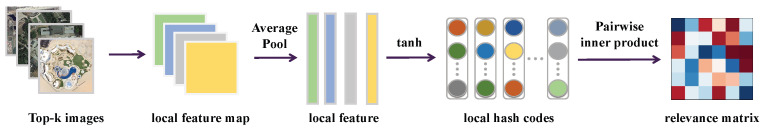
The pipeline for constructing the relevance matrix from retrieved top-k images. Local features are obtained via channel-wise average pooling from convolutional feature maps and transformed into local hash codes using a tanh function. For each segment images, pairwise inner products of hash codes are computed to form a relevance matrix representing mutual similarities.

After training the GHLM, given a query image, we generate a ranking list of images by calculating their Hamming distances. The features from the last convolutional layer of the backbone network are then used to construct the local hash. The process of constructing the relevance matrix is shown in Figure 5. Specifically, given a convolutional feature $g\in R^{H\times W\times C}$ as input, we apply channel-wise average pooling to obtain a local descriptor, and then compute the local hash $h_{l}$ by applying the tanh function:

(11) $$h_{l}^{\left(c\right)}=\tanh\left(\frac{1}{H\times W}\sum_{i=1}^{H}\sum_{j=1}^{W}g_{i,j}^{\left(c\right)}\right).$$

where *c* denotes the *c*-th channel of the input feature *g*, and i,j represent the spatial positions of the feature map $g^{\left(c\right)}$.

After obtaining the local hash, we divide the ranking list into segments of length *L*. The local hashes of the images in each segment are represented as $\left[h_{l}^{1},h_{l}^{2},\ldots ,h_{l}^{L}\right]$, where $h_{l}^{1}$ corresponds to the query image. This allows us to calculate the relevance between the query image and the images in the segment. For each segment, we then construct the relevance matrix $R\in R^{L\times L}$ by calculating the pairwise similarity between the images in the segment, where each element R(i,j) is defined as:

(12) $$R\left(i,j\right)={\left(h_{l}^{i}\right)}^{T}h_{l}^{j}.$$

For each relevance matrix *R*, we define a ground-truth similarity matrix $G\in R^{L\times L}$ to represent the semantic relevance. Specifically, if images $I_{i}$ and $I_{j}$ belong to the same category, then G(i,j)=1; otherwise, G(i,j)=0. The CNN model is trained to predict the similarity matrix *G* from the relevance matrix *R*. To achieve this, we optimize the CNN model by minimizing the following loss function:

(13) $$\min_{\omega }||O-{G||}_{F}^{2}=\min_{\omega }||f\left(R,W\right)-{G||}_{F}^{2},$$

where O=f(R,W) is the output of the CNN model, and *W* denotes the network parameters. The notation $\left|\right|\cdot {\left|\right|}_{F}$ represents the Frobenius norm, which is the square root of the sum of the absolute squares of its elements.

**Figure 6:**
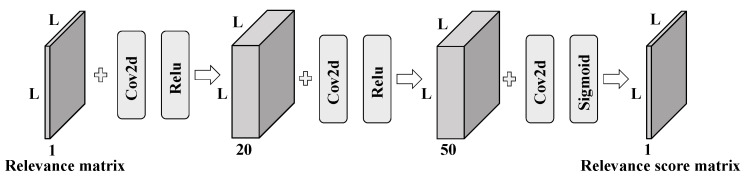
The CNN model used in our LHRM. The input size of the network is determined by the segment length L. The dimensions of each layer are shown in the figure. Each convolutional kernel has a size of 3 × 3 with a stride of 1. ReLU activation is applied after the first two convolutional layers, while the final layer uses a sigmoid function to constrain the output within a range of 0 to 1.

Inspired by [42,43], the framework for the CNN model we design is shown in Figure 6. The network consists of four convolutional layers, each using a 3 × 3 kernel with a padding size of 1 to preserve the spatial dimensions throughout the network. The output dimensions of the four convolutional layers are 20, 50, 50, and 1, respectively. For the first three convolutional layers, we apply the ReLU activation function, while the final layer uses a sigmoid function to normalize the output to a range between 0 and 1. To maintain consistency between the input and output dimensions, no down-sampling operations are included in the network. The input to the network is the relevance matrix *R*, corresponding to the segment in the ranking list, and the output of the final layer is the predicted relevance score matrix *O*, which is optimized to match the ground-truth similarity matrix *G* by minimizing the loss function, as described in Equation (13).

#### 3.3.2. Re-Ranking Process

Through the CNN model, the relevance matrix *R* for the segment is transformed into the relevance score matrix *O*. The elements in the first row and first column of *O* represent the relevance between the query image and the images in the segment, forming the basis for re-ranking. To address the asymmetry in the predicted similarity matrix *O*, we average the values in the first row and first column to more effectively represent the relevance between the query image and the corresponding database images, as suggested by [43]. The relevance score for the *i*-th image in the re-ranking list is then defined as:

(14) $$s_{i}=\frac{O_{1,i}+O_{i,1}}{2},\;i=1,2,3,\ldots ,L.$$

After calculating the relevance score $s_{i}$, the Hamming distance $H_{i}$ for each image $s_{i}$ is weighted by $s_{i}$, and the images within each segment are re-ranked based on this weighted Hamming distance. The weighted Hamming distance is expressed as:

(15) $$H_{w}=\left(1-s_{i}\right)\times H_{i}.$$

## 4. Experiment

### 4.1. Dataset

To evaluate the retrieval performance of our proposed method, we conducted extensive experiments on three widely used remote sensing image datasets: WHU-RS [44], UCMD [45], and AID [46].

#### 4.1.1. WHU-RS

**Figure 7:**
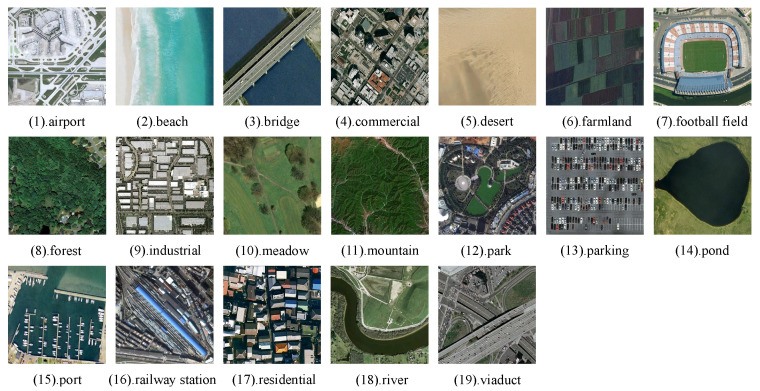
Examples of different categories in the WHU-RS.

The WHU-RS dataset was extracted from Google Earth and contains 19 categories, such as airports, deserts, and forests. Each category includes approximately 50 images, each with dimensions of 600 × 600 pixels. Examples of images from different categories in the dataset are shown in Figure 7.

#### 4.1.2. UCMD

**Figure 8:**
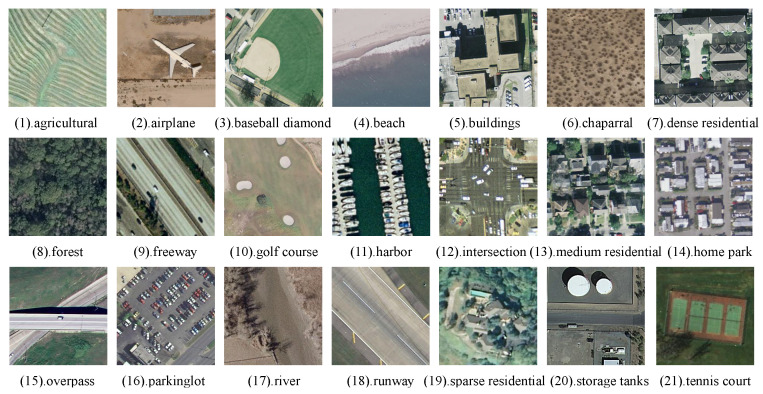
Examples of different categories in the UCMD.

The UCMD dataset was derived from the USGS National Map Urban Area Imagery collection and comprises 21 land cover classes. Each class includes 100 images, with dimensions of 256 × 256 pixels and a spatial resolution of 0.3 m per pixel. Examples of images from different categories in the dataset are shown in Figure 8.

#### 4.1.3. AID

**Figure 9:**
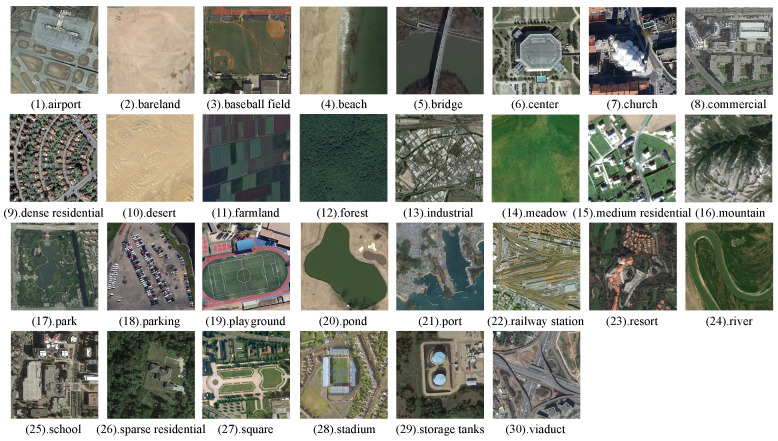
Examples of different categories in the AID.

The AID dataset consists of 30 categories and a total of 10,000 images. Each category includes between 220 and 420 images, all with dimensions of 600 × 600 pixels. The categories represent diverse scenes, such as airports, farms, roads, and rivers. Examples of images from different categories in the dataset are shown in Figure 9.

For the aforementioned datasets, we randomly select 80% of the labeled samples from each class as the training set, with the remaining 20% used as the testing set.

### 4.2. Experimental Settings

The proposed DRH method was implemented using the PyTorch deep learning framework and trained on an NVIDIA GeForce RTX 4090 GPU. For the backbone network in GHLM, Root Mean Squared Propagation (RMSprop) was used as the optimizer during training. The learning rate was set to $1\times 10^{-5}$, with a batch size of 64 and a total of 100 training epochs. Both α and β were set to 0.2, and the margin *m* was set to 0.3. For the CNN model in LHRM, the number of epochs was set to 5, and the segment length *L* was set to 7. All other training parameters were the same as those for GHLM.

### 4.3. Evaluation Metrics

To evaluate the performance of the retrieval methods, we use three metrics: Mean Average Precision (MAP), Recall, and Precision–Recall. These metrics have been commonly used in existing CBRSIR works, such as [13,14], and the definitions of these metrics are as follows:

#### 4.3.1. Mean Average Precision (MAP)

MAP is a widely used performance metric for evaluating image retrieval systems. For each query image, a ranking list is generated by ordering the images in the database based on their Hamming distance from the query image. The retrieval performance for each query is then quantified by computing its Average Precision (AP). MAP is subsequently obtained by averaging the AP scores across all queries. The formula for MAP is expressed as:

(16) $$\mathrm{MAP}=\frac{1}{N_{q}}\sum_{i=1}^{N_{q}}\frac{1}{n_{i}}\sum_{k=1}^{K}P\left(k\right)I\left(k\right).$$

where $N_{q}$ is the total number of query images in the dataset, $n_{i}$ is the number of relevant images for the *i*-th query in the database, P(k) is the precision at position k for the *i*-th query, which is the proportion of relevant images in the top *k* retrieved results, and I(k) is a binary function, where I(k)=1 if the *k*-th image is relevant to the *i*-th query and I(k)=0 otherwise.

#### 4.3.2. Recall

Recall is used to evaluate the effectiveness of a system in retrieving all relevant images for a given query. It measures the proportion of relevant images that are successfully retrieved out of the total number of relevant images available in the database. Recall is defined as:

(17) $$\mathrm{Recall}=\frac{r}{n},$$

where *r* is the number of relevant images retrieved by the system, and n is the total number of relevant images for the query in the database.

#### 4.3.3. Precision–Recall

Precision–Recall is commonly used to evaluate the trade-off between precision and recall in image retrieval systems. Precision measures the proportion of relevant images among the top retrieved results, while recall measures the proportion of relevant images retrieved out of the total number of relevant images in the database. The Precision–Recall curve is constructed by varying the number of retrieved images, with a higher area under the curve indicating better performance.

### 4.4. Retrieval Result

To evaluate the performance of the proposed DRH method, we conduct comparison experiments with eight deep hashing models: DHNN-L2 [11], DPSH [47], FAH [48], DAH [13], AHCL [14], DHCNN [49], SWTH [27], and DGSSH [38]. Table 1 presents the MAP values of different methods on three datasets with varying bit lengths. Please note that bolded terms indicate the best performance in image retrieval. From the table, we can find the following conclusions: (1) The bit length of hash codes significantly impacts retrieval performance. Longer hash bit lengths provide higher discriminative power, enabling more precise representation of image features and improving retrieval accuracy. (2) For simpler datasets such as WHU-RS and UCMD, most methods perform well, but DPSH and SWTH are less effective. For more complex datasets like AID, DPSH, DAH, and SWTH exhibit reduced performance. (3) Across all three datasets and bit lengths, our DRH method consistently achieves the highest MAP values, outperforming the other methods.

**Figure 10:**
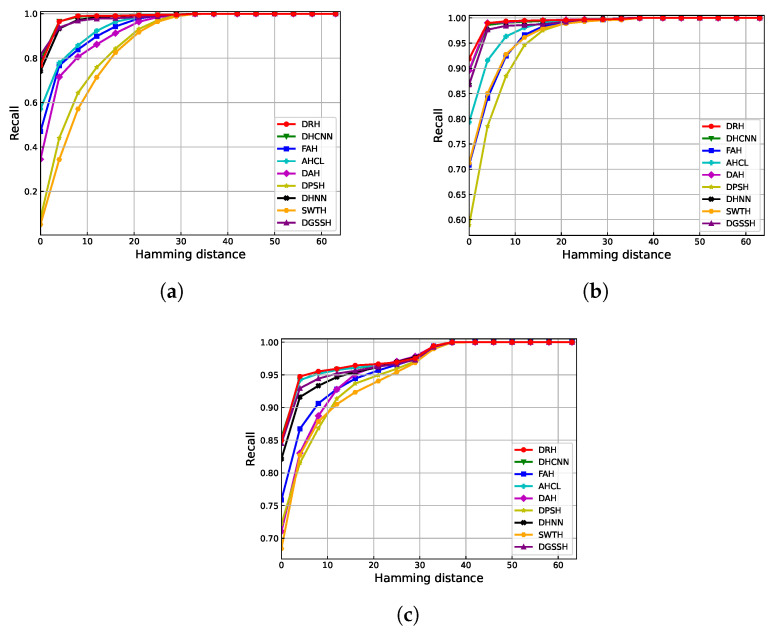
Recall at different Hamming distances for 64-bit hash codes across three datasets: (**a**) WHU-RS, (**b**) UCMD, and (**c**) AID.

**Figure 11:**
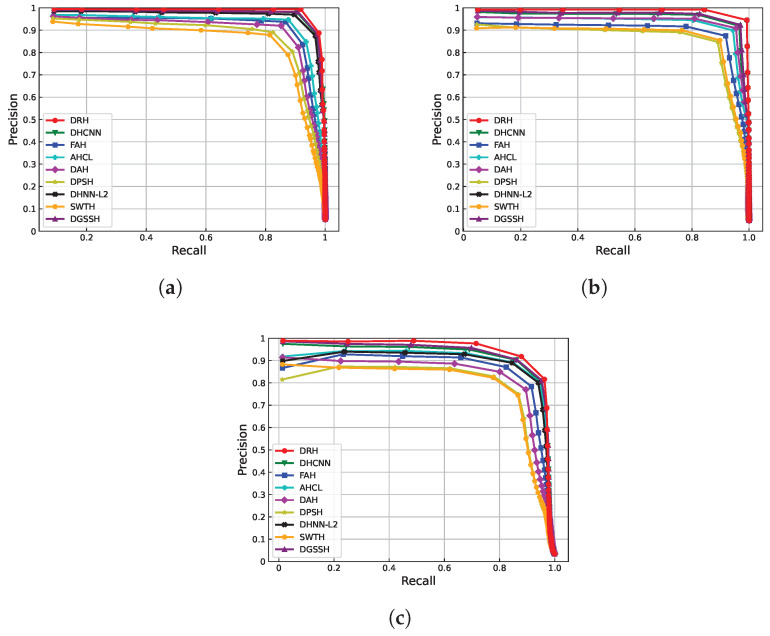
PR curves at 64-bit hash codes for three datasets: (**a**) WHU-RS, (**b**) UCMD, and (**c**) AID.

To further demonstrate the effectiveness of our proposed DRH method, we use two additional evaluation metrics besides MAP: Recall at different Hamming distances and Precision–Recall curves. As shown in Figure 10 and Figure 11, DPSH, DAH, and SWTH exhibit poor retrieval performance. In contrast, our proposed DRH method consistently outperforms the others, achieving better results in both Recall at different Hamming distances and Precision–Recall curves across all datasets. In addition, we quantitatively measure the area under each PR curve (AUC), as reported in Table 2. DRH achieves the highest AUC on all three datasets (0.9338 on AID, 0.9401 on UCMD, and 0.8934 on WHURS-19), further confirming its superior retrieval performance.

### 4.5. Ablation Study

To investigate the contribution of each component to the DRH model, we conducted ablation experiments, focusing on the effectiveness of the key components in enhancing retrieval performance. The DRH model comprises two main components: GHLM and LHRM. GHLM leverages a weighted pairwise similarity loss to guide the learning of hash codes, while LHRM uses a lightweight CNN to refine the retrieval results by re-ranking.

Three variants of the DRH model were constructed:

- DRH-B: Includes only the backbone, hash layer, and classification layer, without any weighting applied to the pairwise similarity loss and without LHRM.
- DRH-R: Adds LHRM to DRH-B, which refines the retrieval results by re-ranking based on local feature analysis.
- DRH-W: Integrates the weighted pairwise similarity loss into DRH-B, allowing the model to focus more on difficult image pairs during training.

The results of these experiments are summarized in Table 3. From the findings, we observe that each component contributes to retrieval performance, with the full DRH model achieving the best results. Taking UCMD as an example, the following conclusions can be drawn:

1. Impact of LHRM: The inclusion of LHRM in DRH-R refines the retrieval process by incorporating local feature re-ranking. This improvement leads to a notable increase in retrieval performance, with MAP increases of 2.12% (16 bit), 1.86% (32 bit), and 1.37% (64 bit).
2. Effect of Weighted Pairwise Similarity Loss: In DRH-B, all image pairs contribute equally to training. However, DRH-W introduces weighting to the pairwise similarity loss, allowing the network to focus on more challenging image pairs. This results in a more discriminative model and yields MAP improvements of 3.22% (16 bit), 3.33% (32 bit), and 3.59% (64 bit) over DRH-B.
3. Combined Impact of Both Components: Both the weighted pairwise similarity loss and LHRM contribute positively to the MAP improvement. The combination of these two components results in the best retrieval performance, surpassing the performance of each component individually.

To evaluate the generalization ability of the proposed DRH framework under different backbone networks, we further conducted experiments using several commonly adopted CNN and Transformer architectures, including AlexNet [50], VGG11 [39], ResNet50 [51], and Swin Transformer [52]. For fair comparison, all models adopt the same hash learning structure and training settings, with only the feature extraction backbone replaced.

As shown in Table 4, the retrieval performance steadily improves with more powerful backbone networks. In particular, VGG11 and Swin Transformer achieve the highest mAP values among the tested models, indicating that the DRH model can effectively benefit from richer and deeper feature representations. However, while Swin Transformer achieves slightly higher accuracy, it comes at the cost of increased retrieval time due to its more complex architecture. In contrast, VGG11 provides a favorable trade-off between accuracy and efficiency, delivering competitive performance with lower retrieval time. Therefore, we adopt VGG11 as the default backbone in our experiments to ensure both effectiveness and efficiency in large-scale retrieval scenarios.

### 4.6. Visualization of Hash Codes and Retrieval Examples

**Figure 12:**
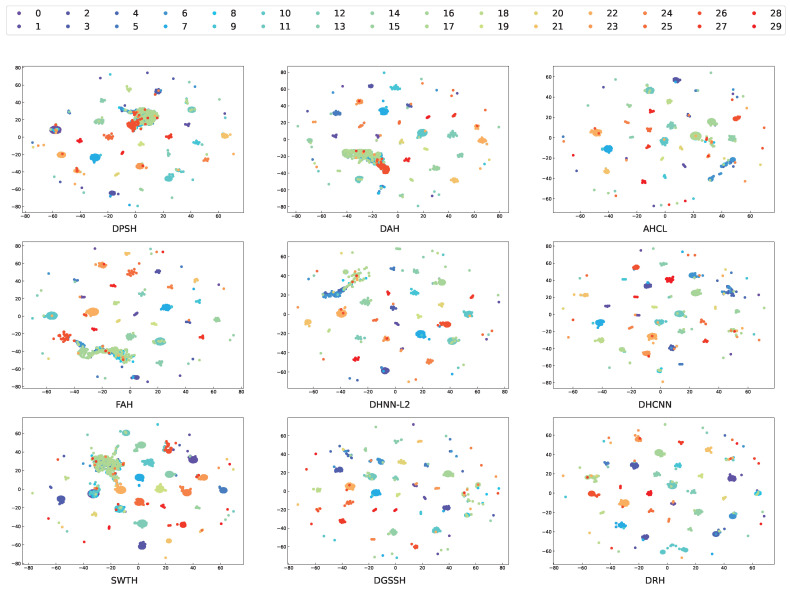
Scatterplots of two-dimensional hash codes obtained via t-sne over the AID dataset. The mappings between the legend IDs and their corresponding categories remain consistent with those in Figure 9.

To analyze and compare the structure and clustering performance of hash codes generated by different methods, we used t-Distributed Stochastic Neighbor Embedding (t-SNE) [53] for dimensionality reduction and visualization on the AID dataset, as shown in Figure 12. The results indicate that DPSH, DAH, FAH, and SWTH exhibit relatively poor clustering performance, with scattered and overlapping clusters. In contrast, the hash codes generated by the GHLM in our proposed DRH method produce more compact clusters with clearer boundaries, therefore demonstrating the effectiveness of the GHLM in capturing discriminative representations.

**Figure 13:**
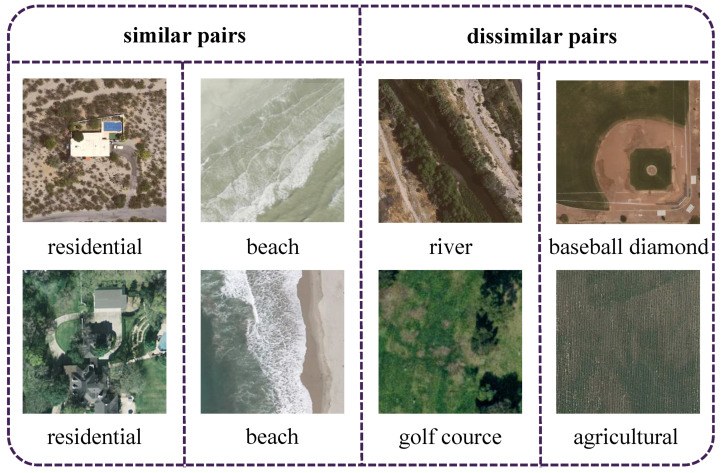
Examples of difficult image pairs from the UCMD dataset: similar pairs with the same label but varying backgrounds (**left**) and dissimilar pairs with different labels but similar backgrounds (**right**).

To evaluate the performance of our proposed GHLM in handling difficult image pairs, we computed the difficulty between query images and database images in the UCMD dataset based on Equation (7). We then selected several difficult image pairs, including two similar pairs and two dissimilar pairs, as depicted in Figure 13. The first two similar pairs share the same label but differ in background or color. The other two dissimilar pairs have different labels but exhibit highly similar backgrounds, which could affect the hash codes generated by the model. Table 5 presents the Hamming distance results between query and database images obtained by different methods. It is important to note that this step was performed using the GHLM method, without the involvement of LHRM. For the similar pairs, our method achieves the smallest Hamming distances, while for the dissimilar pairs, it produces the largest Hamming distances. These results demonstrate the effectiveness of our proposed GHLM in handling difficult image pairs.

**Figure 14:**
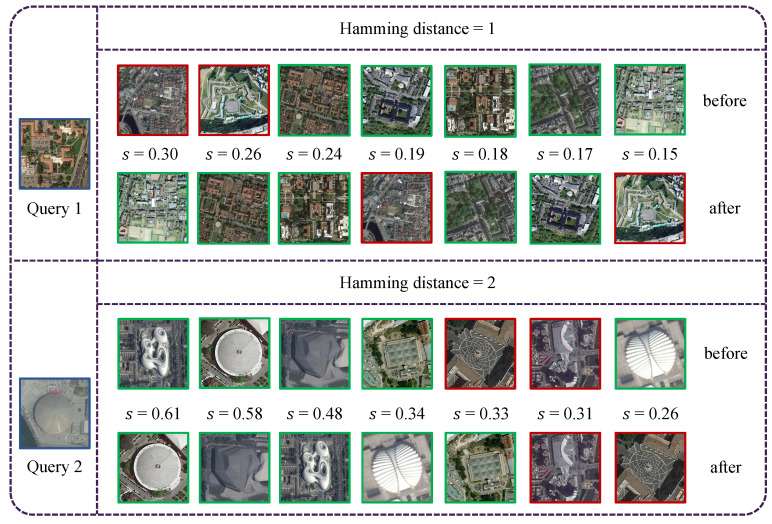
Retrieval examples before and after applying LHRM for two queries from the AID dataset. The left image (blue box) shows the query. The first row presents initial retrieval results, while the second row shows results after applying LHRM. Hamming distance and similarity scores are displayed for each result. Green boxes highlight relevant results, and red boxes highlight irrelevant results. Best viewed in color.

In addition, to verify the effectiveness of LHRM, we present retrieval examples from the AID dataset in Figure 14, where all retrieved images share the same Hamming distance. For each query, we show the results before and after applying LHRM, with relevant images highlighted in green and irrelevant ones in red. The comparison demonstrates that LHRM improves retrieval performance by ranking relevant images higher and pushing irrelevant ones lower.

### 4.7. Hyperparameter Analysis

In this section, we investigate the influence of different parameters in our method. As shown in Equations (4) and (7), there are three key parameters: α, β, and the threshold *m*. The hyperparameters α and β balance the contributions of the pairwise similarity loss $L_{sim}$, classification loss $L_{class}$, and quantization loss $L_{quan}$. The threshold *m* defines a margin for dissimilar image pairs, enabling the model to focus on challenging pairs. For consistency, we set the length of the hash codes to 64 bits and conducted experiments on the UCMD dataset, varying the parameter values from 0 to 1 with a step size of 0.1.

**Figure 15:**
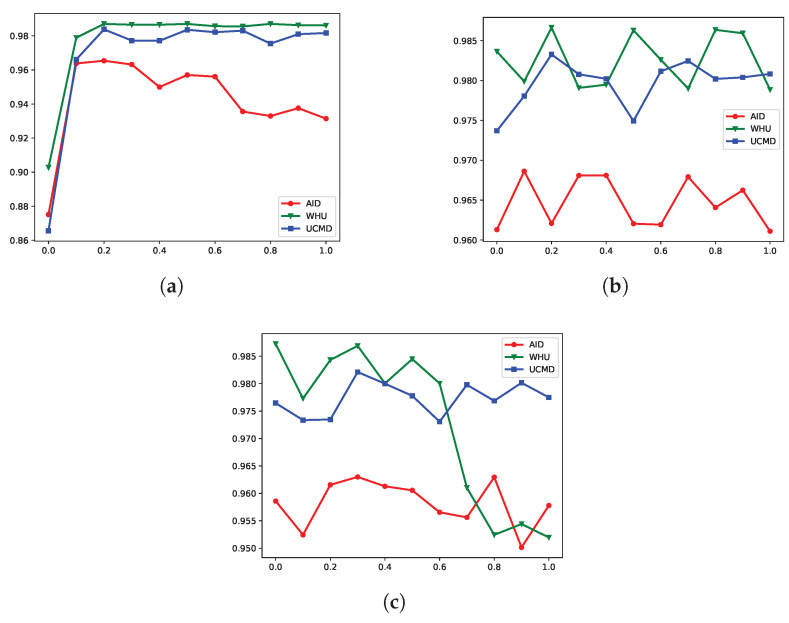
Impact of different hyperparameters: (**a**) α, (**b**) β, and (**c**) *m*.

The hyperparameter experiment results are shown in Figure 15. From Figure 15a, it can be seen that the retrieval performance improves as the value of α increases and stabilizes after reaching 0.2. When α is set to 0, the classification loss is entirely removed, leading to a significant drop in MAP scores. This demonstrates the importance of incorporating semantic information in enhancing retrieval performance. As shown in Figure 15b, β has a small influence on the results, showing slight variations across datasets. Given this, we set β to 0.2, consistent with the value chosen for α. From Figure 15c, it is evident that the margin *m* influences the results to some extent. For the AID and UCMD datasets, the changes are relatively small, showing stable performance. In contrast, the WHU dataset exhibits more noticeable variations with varying values of *m*. We chose *m* to be 0.3, as it achieves relatively high accuracy across all three datasets.

### 4.8. Retrieval Efficiency Analysis

In addition to retrieval accuracy, retrieval efficiency is also a critical factor for image retrieval systems. Table 6 presents a comparison of the training and retrieval times for six methods, including AHCL, DPSH, DAH, SWTH, DGSSH, and DRH, under different hash code lengths on the AID dataset.

In the training phase, AHCL and DGSSH achieve the shortest training times due to their asymmetric learning approach. DPSH and DAH exhibit similar training times. SWTH and DRH have the longest training times, with SWTH requiring more time due to the computational complexity of the Swin–Transformer backbone, while DRH takes longer because both the GHLM and the CNN model in the LHRM need to be trained. However, since the training process is conducted offline and only performed once, the overall time consumption remains acceptable.

In the retrieval phase, DPSH, AHCL, and DAH exhibit similar performance. SWTH and DGSSH have longer retrieval times due to the Swin–Transformer backbone, while DRH also requires a similar retrieval time because of the additional computation introduced by LHRM. However, considering the improvement in accuracy, the retrieval time is still acceptable.

## 5. Conclusions

To address the challenges of imbalanced training between easy and difficult image pairs, as well as the confusion in distinguishing images with the same Hamming distances but different categories, we propose a novel DRH method for remote sensing image retrieval. First, we design a GHLM to extract representative features from remote sensing images and generate initial ranking results. A weighted pairwise similarity loss is introduced to prioritize difficult image pairs based on global feature similarity during training. Next, we design an LHRM to distinguish images sharing the same Hamming distance but belonging to different categories, thereby further improving retrieval performance. Extensive experiments on three benchmark remote sensing datasets demonstrate that our method outperforms existing approaches.

Despite the promising results, our method still has certain limitations. The current weighting strategy in GHLM relies on global feature similarity, which may not fully capture fine-grained differences in complex scenes. Moreover, the LHRM involves additional computational cost during the re-ranking stage, which could affect the retrieval speed in real-time applications.

In the future, we plan to develop more advanced methods for better feature extraction and to further improve retrieval performance. Specifically, we will investigate triplet-based optimization strategies to better capture the relative similarity relationships among visually similar but semantically distinct images, thereby enhancing the model’s ability to distinguish these challenging pairs. Besides remote sensing image retrieval, our proposed GHLM and LHRM also possess the potential to generalize to other image-related tasks, such as social network classification [54] and scene classification [55,56], where learning discriminative structural or topological information is equally important. 

## Figures and Tables

**Table 1:** Image retrieval results in terms of MAP (%) with 16, 32, and 64 hash bits on three datasets.

Methods	WHU-RS			UCMD			AID		
	16 Bit	32 Bit	64 Bit	16 Bit	32 Bit	64 Bit	16 Bit	32 Bit	64 Bit
DHNNs-L2 [11]	94.25	96.34	97.76	92.29	94.72	95.03	83.31	86.19	93.15
DPSH [47]	86.67	90.87	92.37	82.24	91.26	92.97	81.96	84.22	87.68
FAH [48]	91.78	95.19	96.74	91.23	94.88	95.14	84.51	91.51	93.33
DAH [13]	94.15	95.99	96.32	86.79	96.27	96.57	81.01	86.37	87.58
AHCL [14]	93.47	94.62	97.32	94.39	95.23	95.66	86.67	91.71	94.07
DHCNN [49]	96.27	97.92	98.31	95.70	95.64	96.34	87.54	92.22	95.85
SWTH [27]	86.67	87.87	90.37	82.71	84.05	87.52	78.91	86.73	87.15
DGSSH [38]	96.66	96.62	97.64	96.32	97.13	98.47	87.17	93.31	95.61
DRH	97.14	98.46	98.73	97.73	97.94	98.72	88.35	94.68	96.21

**Table 2:** AUC values of PR curves on different datasets.

Method	AID	UCMD	WHURS-19
DRH	0.9338	0.9401	0.8934
DHCNN	0.9068	0.9122	0.8851
FAH	0.8563	0.8513	0.8330
AHCL	0.8894	0.8861	0.8445
DAH	0.8212	0.8936	0.8152
DPSH	0.7845	0.8177	0.7950
DHNN-L2	0.8832	0.9301	0.8758
SWTH	0.7787	0.8259	0.7718
DGSSH	0.9171	0.9184	0.8852

**Table 3:** Ablation experiments on different datasets in terms of MAP (%) with component configurations.

Methods	Weighted Loss	Reranking	WHU-RS			UCMD			AID		
			16 Bit	32 Bit	64 Bit	16 Bit	32 Bit	64 Bit	16 Bit	32 Bit	64 Bit
DRH-B	x	x	92.08	96.14	96.52	94.43	94.43	95.02	85.11	92.62	93.02
DRH-R	x	✓	92.52	96.85	96.91	96.55	96.29	96.39	86.23	92.88	94.17
DRH-W	✓	x	96.77	98.43	98.59	97.65	97.76	98.61	88.21	93.41	95.33
DRH	✓	✓	97.14	98.46	98.73	97.73	97.94	98.72	88.35	94.68	96.21

**Table 4:** Retrieval performance with different backbone networks.

Backbone	mAP (%)	Retrieval Time (s)
AlexNet [50]	84.72	12.37
ResNet50 [51]	88.33	12.45
VGG11 [39]	92.25	12.43
Swin–Transformer [52]	92.41	13.56

**Table 5:** Hamming distances for selected difficult pairs across different methods.

Methods	Similar		Dissimilar	
	Pair 1	Pair 2	Pair 1	Pair 2
AHCL [14]	32	31	32	31
DAH [13]	32	34	18	31
DHNN-L2 [11]	33	32	31	26
DHCNN [49]	31	30	34	26
FAH [48]	32	35	32	31
DPSH [47]	34	33	14	30
SWTH [27]	35	34	16	25
DGSSH [38]	30	29	35	33
DRH	29	28	37	35

**Table 6:** Time comparison (in seconds) for different hashing methods.

Methods	Training Time			Retrieval Time		
	16 Bit	32 Bit	64 Bit	16 Bit	32 Bit	64 Bit
DPSH [47]	1221.41	1231.21	1240.89	11.41	11.82	12.16
AHCL [14]	946.31	965.37	986.82	11.10	11.25	11.42
DAH [13]	1234.91	1236.32	1284.45	12.55	12.63	12.88
SWTH [27]	1336.31	1346.24	1334.61	13.22	13.44	13.42
DGSSH [38]	975.51	971.52	1019.86	13.17	13.38	13.46
DRH	1398.36	1403.14	1404.23	13.23	13.27	13.36

## Data Availability

Data are contained within the article.
